# Reconfiguration from emergency to urgent elective neurosurgery for glioblastoma patients improves length of stay, surgical adjunct use, and extent of resective surgery

**DOI:** 10.1093/nop/npac034

**Published:** 2022-05-02

**Authors:** Rosa Sun, Shivam Sharma, Helen Benghiat, Sara Meade, Paul Sanghera, Gregory Bramwell, Santhosh Nagaraju, Ute Pohl, Camilla Dawson, Vladimir Petrik, Ismail Ughratdar, Anwen White, Athanasios Zisakis, Satheesh Ramalingam, Vijay Sawlani, Colin Watts, Victoria Wykes

**Affiliations:** Department of Neurosurgery, University Hospitals Birmingham, Birmingham, UK; Department of General Surgery, Royal Wolverhampton NHS trust, UK; Hall-Edwards Radiotherapy Research Group, Cancer Centre, Queen Elizabeth Hospital, Birmingham, UK; Hall-Edwards Radiotherapy Research Group, Cancer Centre, Queen Elizabeth Hospital, Birmingham, UK; Hall-Edwards Radiotherapy Research Group, Cancer Centre, Queen Elizabeth Hospital, Birmingham, UK; Department of Neurosurgery, University Hospitals Birmingham, Birmingham, UK; Department of Cellular Pathology, University Hospitals Birmingham, Birmingham, UK; Department of Cellular Pathology, University Hospitals Birmingham, Birmingham, UK; Department of Speech and Language, University Hospitals Birmingham, Birmingham, UK; Department of Neurosurgery, University Hospitals Birmingham, Birmingham, UK; Department of Neurosurgery, University Hospitals Birmingham, Birmingham, UK; Department of Neurosurgery, University Hospitals Birmingham, Birmingham, UK; Department of Neurosurgery, University Hospitals Birmingham, Birmingham, UK; Department of Neuroradiology, University Hospitals Birmingham, Birmingham, UK; Department of Neuroradiology, University Hospitals Birmingham, Birmingham, UK; Department of Neurosurgery, University Hospitals Birmingham, Birmingham, UK; Institute of Cancer and Genomic Sciences, University of Birmingham, Birmingham, UK; Institute of Cancer and Genomic Sciences, University of Birmingham, Birmingham, UK

**Keywords:** glioblastoma, health service, Neuro-oncology, neurosurgery, oncology practice

## Abstract

**Background:**

Glioblastoma (GB) is the most common intrinsic brain cancer and is notorious for its aggressive nature. Despite widespread research and optimization of clinical management, the improvement in overall survival has been limited. The aim of this study was to characterize the impact of service reconfiguration on GB outcomes in a single centre.

**Methods:**

Patients with a histopathological confirmation of a diagnosis of GB between 01/01/2014 and 31/12/2019 were retrospectively identified. Demographic and tumour characteristics, survival, treatment (surgical and oncological), admission status, use of surgical adjunct (5-aminolevulinic acid, intra-operative neuro-monitoring), the length of stay, extent of resection, and surgical complications were recorded from the hospital databases.

**Results:**

From August 2018 the neurosurgical oncology service was reconfigured to manage high-grade tumours on an urgent outpatient basis by surgeons specializing in oncology. We demonstrate that these changes resulted in an increase in elective admissions, greater use of intra-operative adjuncts resulting in the improved extent of tumour resection, and a reduction in median length of stay and associated cost-savings.

**Conclusions:**

Optimizing neuro-oncology patient management through service reconfiguration resulted in increased use of intra-operative adjuncts, improved surgical outcomes, and reduced hospital costs. These changes also have the potential to improve survival and disease-free progression for patients with GB.

Gliomas are the most common primary tumours of the central nervous system (CNS). The estimated annual incidence is 6.6 per 100,000 individuals in the USA^1^ and is predicted to rise to 22 per 100,000 by 2035. In the UK during 2016, there were 5250 deaths from brain tumours; this represents 14 deaths per day.^2^ Despite a worldwide improvement in the five-year net survival for most CNS cancers over the last two decades,^3^ malignant CNS tumours still hold the poorest prognosis and are responsible for the highest number of potential years of life lost amongst all cancers (mean 20 years).^4^ Approximately half of all newly diagnosed gliomas are classified as World Health Organization (WHO) grade IV glioblastoma (GB), the most malignant type of brain cancer. The median expected survival without treatment is less than three months.^5^ Factors that are associated with more a favourable prognosis include young age, good performance status, location of tumour,^6^ and several molecular markers such as isocitrate dehydrogenase 1 and 2 mutations^7^ and O6-methylguanine-DNA methyltransferase methylation status.^8^

The gold standard treatment for GB combines surgical resection, radiotherapy, and chemotherapy, with the aim of slowing tumour progression and improving patient survival.^9^ Despite decades of refinement, this approach results in a median survival time of 14.6 months.^9^ Currently, the alkylating agent temozolomide remains the first line, and one of the few effective chemotherapy agents available for patients with glioblastoma. There is an urgent need to identify novel agents. Over the last decade, significant advances in brain tumour imaging, intraoperative technologies, and neurosurgical techniques have been achieved with the aim of maximizing extent of resection (EOR) whilst improving patient safety and preventing post-operative neurological deficit. A growing body of clinical data supports the prognostic importance of gross total resection for patients with GB,^10,11^ and this is being incorporated into European guidelines for the management of patients with GB.^12–14^

In addition to optimizing current treatment modalities for patients with GB, modifying how the treatment is delivered via service reconfiguration can positively impact patient outcomes.^15^ The “*Getting It Right First Time*” (GIRFT) programme has examined in detail the way that cranial neurosurgery is provided in England. It set out to identify differences in procedures and practice, as well as common issues. A review of neuro-oncology practice across the 24 trusts in England, identified that “*elective spells*” are, on average, seven days shorter than non-elective spells.^16^ A myriad of factors can contribute to the longer length of stay when admitted via an emergency pathway including awaiting inpatient imaging investigations, pausing of antiplatelet/anti-coagulant medication before surgery to reduce bleeding risk, neurosurgical emergency operations taking priority over neuro-oncology, and pressure to complete cases urgently due to pressures in the emergency list and discharge planning. Consequently, one of the key recommendations of GIRFT for glioma management was to increase the proportion of urgent elective admissions.

The neuro-oncology service at Queen Elizabeth Hospital Birmingham covers a large catchment area with a population of 3.6 million within the West Midlands and captures oncology practice spanning eight different hospital sites. During the six-year period from 01/01/2014 to 31/12/2019, a gradual service reconfiguration has been achieved at our hospital. We transitioned from a predominantly emergency based admission system to a predominantly urgent planned elective admission; from operating on patients on the next available emergency list to planned dedicated oncology lists led by specialist neurosurgical oncology consultant neurosurgeons as defined by National Institute for Clinical Excellence (NICE) guidelines in 2006.^17^ Here we review how this service reconfiguration, coupled with the introduction of surgical adjuncts to the neurosurgical armamentarium has impacted on elective admissions, use of dedicated oncology lists, the extent of surgical resection, post-operative complications and hospital LOS.

## Methods

The patient cohort was identified retrospectively from a search of the department of pathology database between 01/01/2014 and 31/12/2019 (six years inclusive), for a confirmed histological diagnosis of GB defined as glioblastoma, gliosarcoma, or glioma (WHO grade IV). Cases were limited to adults (16 years old), and patients who had transformed from a lower grade glioma were excluded. Cases of re-do operations, whereby the primary diagnostic procedure was completed prior to the study period, were also excluded.

Data were collected up to 02/02/2021 from available hospital records for demographics, admission status, length of stay (LOS), procedure (biopsy or craniotomy), oncology treatment, use of intra-operative adjunct, complications, the extent of resection, and survival. Sources of data included clinical letters, multi-disciplinary team (MDT) notes, operation notes, radiological reports, procurement of 5-aminolevulinic acid (5-ALA), and use of intra-operative neurophysiological monitoring reports. Post-operative MRIs were reviewed in cases of equivocal or ambiguous radiological reporting regarding the extent of resection.

### Outcomes Related to Elective and Emergency Admission Status

Patients with a diagnosis of a GB were identified and their admission pathway (emergency versus elective), what type of surgery (biopsy versus craniotomy), and median length of stay were recorded. Thirty-day post-operative complications and extent of resection for patients undergoing craniotomies were described and compared between patients admitted through elective or emergency pathways. EOR is classified by the first post-operative MRI obtained within 72 hours as per NICE guidelines^14^ with the following definitions: complete resection of enhancing tumour (CRET), gross total resection (GTR; >90% resection), or subtotal resection (STR; <90% resection).

### Outcomes Related to Surgical Management

The use of neurosurgical adjuncts recorded included 5-ALA and intra-operative neuro-monitoring. The latter group includes awake with speech and motor monitoring, awake with speech monitoring alone, awake with motor monitoring alone, or asleep with motor monitoring.

The extent of resection was recorded and grouped into those who received 5-ALA and those who did not. The difference in post-operative neurological deficit between those who received intra-operative neuro-monitoring and those who did not was measured.

### Post-operative Treatment

Patients were separated into groups depending on whether they underwent biopsy or craniotomy; oncological treatment categories were divided into full Stupp regime (60 gray in 30 fractions with concomitant Temozolomide; followed by six months of adjuvant Temozolomide); palliative radiotherapy alone; or no treatment. Survival was characterized by the Kaplan–Meier method.

### Statistical Analyses

Data analysis was performed using Excel and SPSS (IBM) version 27. Continuous variables were reported using medians and interquartile ranges due to the non-normality of the data and categorical variables were reported as numbers and percentages. All pairwise comparisons of categorical data were analyzed using Pearson’s chi-squared test. Survival data were compared across groups using independent samples median testing with Yate’s correction for continuity. Estimations of 95% confidence intervals were calculated using independent-samples Hodges-Lehman median difference estimator. Clinical significance was defined at the level of *P* < 0.05.

### Ethics Approval and Consent to Participate

This study was registered as a qualitative improvement study/audit at the Queen Elizabeth Hospital Birmingham Research and Audit department and no ethical approval was required.

## Results

Between 01/01/2014 and 31/12/2019, 610 adult patients (63.3% male) underwent primary surgery with confirmed histopathological tissue diagnosis of glioblastoma WHO grade IV. Of these, two patients were lost to follow-up and were not included in the analysis. The median age at the time of surgery was 63 years (range 16–89 years).

A total of 349 patients underwent oncology treatment at Queen Elizabeth Hospital Birmingham, UK. 199 underwent oncology treatment in district general hospitals, and 9 patients underwent oncology treatment outside of our catchment area. For 51 patients, we were unable to obtain geographical information on their oncology treatment.

For patients with GB undergoing surgery, biopsy cases constituted 33.2% and craniotomies 66.8% of all cases. The median age of patients undergoing biopsy was 5.6 years older than craniotomy (67.4 vs. 61.8 years respectively). Frontal and temporal lobes were the most affected locations, with overall equal distribution between left and right cerebral hemispheres. Demographic data are summarized in Table 1.

**Table 1.** Demographics and supra-tentorial location of patients with glioblastoma operated at a single neurosurgical centre between 01/01/2014 and 31/12/2019.

Demographics (*N* = 608)			
Gender			
Male		385 (63.3%)	
Female		223 (36.7%)	
Age (years)			
	Median age	Range	Number
Biopsy	67.4	16.8–83.3	144
Craniotomy	61.8	16.1–88.6	464
Anatomical location of GB			
Frontal			171
Temporal			159
Parietal			63
Occipital			39
Cortical (crossing multiple lobes)			108
Multifocal			49
Isolated thalamic			9
Intraventricular			4
Isolated insular			3
Isolated brainstem			2
Cerebellar			1

### Outcomes Stratified by Elective Versus Emergency Admission Status

**Figure 1.**
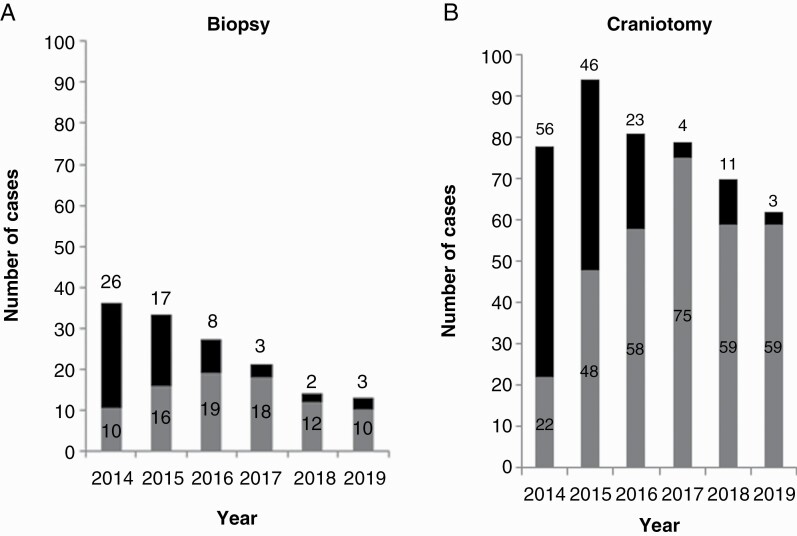
Total annual number of biopsy (a) or craniotomy (b) procedures, tabulated by operating theatre status: Emergency list (black) or elective list (grey).

An overview of the total annual biopsy and craniotomy procedures performed on either an elective or emergency operative theatre list is provided in Figure 1. In total, 144 patients underwent biopsy (85 elective, 59 emergency) and 464 craniotomy (321 elective, 143 emergency). Between 2014 and 2019, the percentage of patients operated on via an urgent elective pathway increased from 28.1% to 92.0% (2014 vs. 2019, *χ*^2^ = 165.6, *P* <0.001), and the proportion of craniotomy procedures increased from 68.4% to 82.7% (2014 vs. 2019, *χ*^2^ = 4.78, *P* = 0.041).

**Figure 2.**
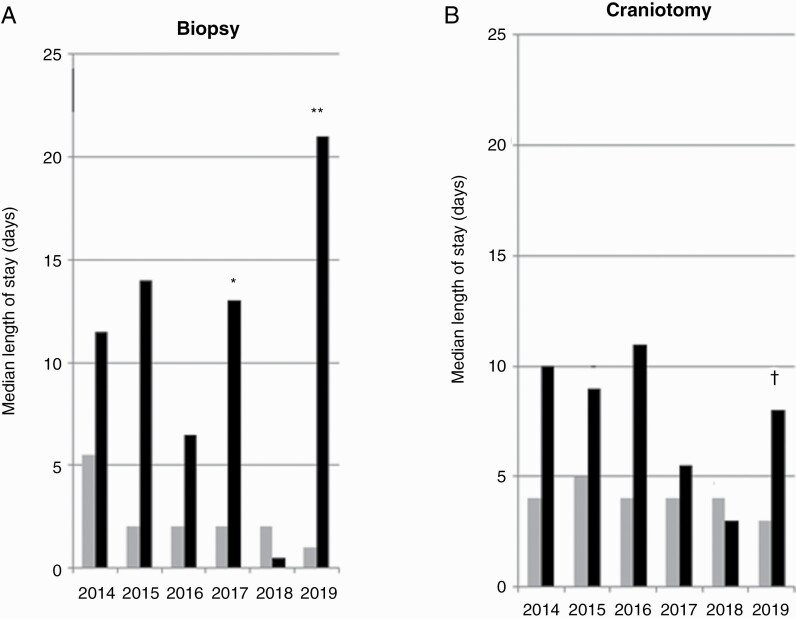
The median length of hospital stay (days) for patients with glioblastoma undergoing first diagnostic procedure by admission status: Patients underwent biopsy (a) or craniotomy (b), on either the urgent elective (grey) or emergency operative theatre list (black). There were limited number of patients who had *emergency biopsy in 2017 (*n* = 2), **emergency biopsy in 2019 (*n* = 3), ^†^emergency craniotomy in 2019 (*n* = 3).

Overall, the median LOS was shorter for elective admissions across both procedures (3 vs. 10 days, *χ*^2^ = 145.17, *P* < 0.0001) as illustrated in Figure 2. For patients on the urgent elective pathway for biopsy, the median LOS was 2 days, whereas for patients on the emergency pathway the median LOC was 10.5 days (*χ*^2^ = 41.03, *P* < 0.0001). In contrast, for patients on the urgent elective pathway receiving craniotomy, without post-operative complication, the median LOS was 4 days, whereas for those on the emergency pathway the median LOS was 9 days (*χ*^2^ = 70.68, *P* <0.0001). Between 2014 and 2019, the median LOS for all procedures was reduced from 9 days to 3 days (*χ*^2^ = 27.00, *P* < 0.001). For biopsies, this fell by 4.5 days (5.5 days in 2014 vs. 1 day in 2019, *χ*^2^ = 9.90, *P* = 0.005). For craniotomies, this fell by 1 day (4 days in 2014 vs. 3 days in 2019, *χ*^2^=0.868, *P* =0.497).

Thirty-day readmission and post-operative complication as per elective or emergency operating list status are summarized in Supplementary Table 1. Thirty-day readmission rates were 1.2% and 6.8% for biopsied patients admitted through elective and emergency pathways, respectively (*χ*^2^ =3.261, *P* = 0.071). For craniotomy patients, readmission rates were 5.6% and 8.4%, respectively (*χ*^2^ =1.268, *P* = 0.261).

Five deaths in total within 30 days of admission occurred, 3 (0.7%) from the elective pathway and 2 (1.0%) from the emergency pathway. One death was caused by post-operative surgical hematoma after elective admission for craniotomy. Three deaths occurred within 30 days due to rapid disease progression and deterioration in clinical status (2 of these patients were admitted through the emergency pathway and one via the elective pathway). Overall complication rates for elective and emergency admissions were 8.6% and 8.9%, respectively (*χ*^2^ = 0.012, *P* = 0.967).

### Outcomes Stratified by 5-ALA and Intra-operative Monitoring Use

The use of 5-ALA and neuro-monitoring adjuncts for patients with GB is summarized in Supplementary Table 2. Adoption of adjunct use began with 3 cases in 2017 (3.8% of total), and increased to become routine with 42 cases in 2019 (67.8% of total). Over this timeframe, intra-operative neuro-monitoring for craniotomy increased from 2 cases in 2017 (2.6%), to 17 cases in 2019 (27.4%).

The use of 5-ALA was associated with an improved extent of resection (CRET and GTR versus STR, *χ*^2^ =24.430, *P* < 0.0001). Of the 56 patients who received 5-ALA, 16 (28.6%) achieved CRET, 31 (55.4%) achieved GTR, and 9 (16.1%) had STR, whereas among those who did not receive 5-ALA (*N* = 408), 54 (13.2%) achieved CRET, 145 (35.5%) GTR, and 209 (51.2%) STR. There was no significant difference in post-operative neurological deficits following intra-operative monitoring (*χ*^2^ =0.309, *P* = 0.579), but this may be partly due to the small number of adverse outcomes. In those who received intra-operative monitoring (*N* = 31), 1 (3.2%) had a post-operative neurological deficit, whereas in those who did not (*N* = 577), 32 (5.6%) suffered post-operative neurological deficit (*χ*^2^ =0.309, *P* = 0.579).

**Figure 3.**
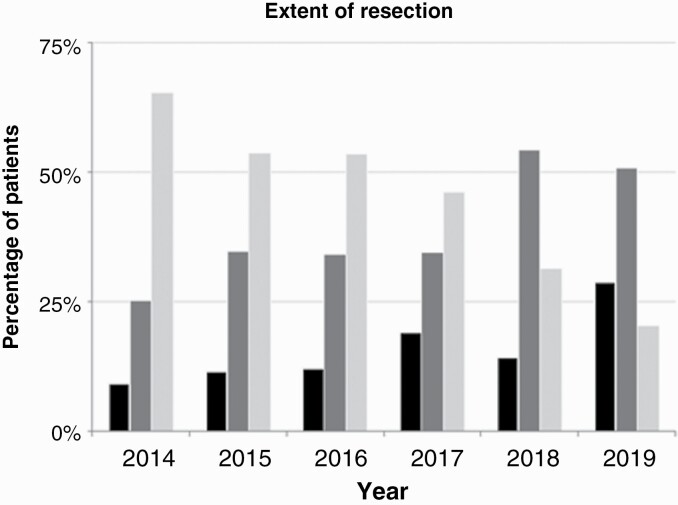
Annual percent of glioblastoma patients ranked as per extent of resection of glioblastoma at first post-operative imaging: Complete resection of enhancing tumour (black); Gross total resection (>90% resection; dark grey), subtotal resection (<90% resection; light grey.

Between 2014 and 2019, EOR for patients who underwent resective surgery has improved from 9.3% CRET in 2014 to 28.8% CRET in 2019, 25.3% GTR in 2014 to 50.9% in 2019, as illustrated in Figure 3.

### Survival Outcomes

**Figure 4.**
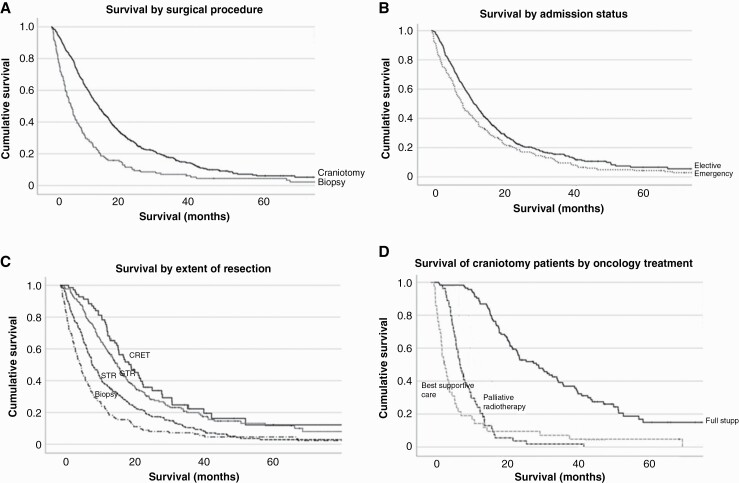
(a) Overall survival of patients with glioblastoma undergoing biopsy (dashed) or craniotomy (solid) at presentation to a single neurosurgical centre between 01/01/2014 and 31/12/2019. (b) Craniotomy for GB as per operating list status: elective and emergency. (c) Extent of resection: complete radiological resection of enhancing tumour, gross total resection, subtotal resection, biopsy. (d) Overall survival of craniotomy patients followed by full Stupp protocol, palliative radiotherapy alone, or best supportive care.

At last survival data collection (02/02/2021), 64 (10.5%) patients were still alive, and the median survival for all patients was 9.5 months (IQR 4.7–17.7 months, SE = 0.7). A Kaplan–Meyer survival curve of patients with GB undergoing either a biopsy or craniotomy is illustrated in Figure 4a.

Median survival was 10.4 months for all patients admitted through the elective pathway and 8.4 months for patients admitted through the emergency pathway, as illustrated in Figure 4b.

Mantel-cox log-rank comparison of overall survival between elective and emergency admissions demonstrates a significant difference in survival (*χ*^2^= 4.977, *P* = 0.032). However, for craniotomies alone, no difference in overall survival was found between elective and emergency admissions (*χ*^2^ = 0.751, *P* = 0.386). For biopsies alone, no difference in overall survival was found between elective and emergency admission (*χ*^2^ = 2.709, *P* = 0.100).

Stratified by EOR (Figure 4c), median survival was CRET 15.9 months, GTR (>90% resection) 13.6 months, STR 8.9 months, and biopsy 5.5 months. Overall, for patients undergoing craniotomies, EOR has a significant effect on survival (*χ*^2^ = 26.353, *P* < 0.0001). Pair-wise comparison of survival by CRET versus GTR (*χ*^2^ = 0.660, *P* = 0.416), GTR versus STR (*χ*^2^ = 12.881, *P* < 0.0001), STR versus biopsy alone (*χ*^2^ = 13.846, *P* < 0.0001). Our results indicate progressive survival advantage offered by higher EOR up to the level of GTR (>90% resection).

Patients who underwent surgical resection and were able to tolerate completion of the Stupp protocol had a median survival of 22.0 months (range = 2.40–89.55; *n* = 85). In comparison, in patients who underwent a biopsy followed by the Stupp protocol, the median survival was 19.7 months (range = 3.62–40.70; *n* = 11). Palliative radiotherapy only regimes in our cohort include 40 Grays in 15 fractions, and 30–34 Grays in 8–10 fractions, and variations of these regimes are dependent on tumour location, performance status, and age of the patient. For patients receiving palliative radiotherapy only, following surgical resection median survival was 7.9 (range = 2.89–41.55; *n* = 53) months while following biopsy only median survival was 6.1 months (range = 1.81–29.32; *n* = 29). For patients receiving best supportive care median survival was 2.8 months (range = 0.85–37.64; *n* = 29). Kaplan–Meier for patients who underwent surgical resection, categorized by oncology treatment is illustrated in Figure 4d.

## Discussion

From 2014 to 2019, a gradual restructuring of the neurosurgical oncology service from an emergency-delivered GB service to an urgent elective service led by specialist consultant neurosurgeons led to an increase in the number of craniotomies achieving maximal resection and access to planned intra-operative adjuncts. There is a trend toward the improved extent of resection and reduced hospital LOS consistent with the literature.

A gradual change in the delivery of neuro-oncology services has been achieved at our trust. Previously, there was a predominance of emergency-based services for patients with GB; transfers from district hospitals at the initial point of presentation were common. Through such a pathway, unnecessary delays caused by the lack of neurosurgical beds in the initial assessment of the patient were routine. Patients with GB may be assessed by non-oncological specialists or the neurosurgical registrar on call. The nature of the emergency operating lists is to prioritize life-threatening emergencies. Operating on urgent elective oncology cases on the emergency list is prone to cancellation due to the time-critical nature of a general neurosurgical emergencies.

Data from patients with GB overwhelmingly supports the fundamental principle of neurosurgical oncology that safe, maximal tumour resection improves symptom control, quality of life, and both progression-free and overall survival.^11^ This study was performed during the period when the ethos regarding surgical management of patients with GB, was also evolving towards maximal safe tumour resection, and our data reflects this trend with a reducing number of biopsy procedures. The increased use of dedicated urgent elective theatre ensures all patients were discussed in the neuro-oncology MDT, with a discussion of optimal surgical adjuncts, and research opportunities prior to review in the clinic within 48 h of the MDT. Currently, our acute neuro-oncology clinic service consists of a one-stop-shop model with assessment by a neurosurgical consultant, review with additional oncology and or neurology epilepsy consultants if required, and informed consent for surgical operation and or participation in research trials, platform studies, and research tissue donation. Meeting with the specialist oncology nurse practitioners at every clinical interaction and provision of a written care plan at the end of the clinical appointment with nurse specialist contact details allows patients to feel supported and have a point of contact for further questions. Similarly, the access to a speech and language therapist within these clinics for those patients who have dysphasia allows for a prompt diagnosis and effective implementation of rehabilitation plans. Access to a same day pre-operative assessment clinic facilitates optimization of patients’ functional status before surgery, and enhancement of post-operative recovery through planned admissions. In addition, same day volumetric imaging for intra-operative neuro-navigation is greatly beneficial for patients so they can get all their investigations and reviews completed on the same day, especially if they are travelling from the farthest areas of our catchment area. Post-operatively, telephone or face-to-face consultation^18^ for tissue diagnosis and oncology treatment plan is discussed with the patient at this dedicated oncology clinic with the input of specialist oncology nurses, maintaining continuity between neurosurgical and oncological care. This clinical structure supports consultant-led, multi-disciplinary patient-focused, and research-orientated delivery of neurosurgical oncology practice.

The urgent elective oncology pathway has reduced pressure on emergency theatres, which not only frees up space for life-saving emergencies, but also reduces the operating time pressure for tumour resections. The elective approach has facilitated the use of neurosurgical adjuncts including 5-ALA for maximal safe resection, through planning for the timing of oral administration 2–4 h before surgery, as well as allowing planning and co-ordination of intra-operative neurophysiological monitoring. Since 2019, we have also introduced the routine use of the surgical aspirator collection pot for the collection of tumour aspirates, which can be beneficial for diagnosis or research purposes.^19^ As per NICE recommendations, patients with suspected high-grade glioma undergoing initial surgery with the potential for complete resection should be offered 5-ALA.^14^ The true benefit of neuro-monitoring has been difficult to assess in the literature, as the preservation or damage of motor or language functions should be taken on a case-by-case basis. Neuro-monitoring has become increasingly popular amongst neurosurgeons for its ability to provide direct and intraoperative guidance for decisions regarding the balance between oncological and functional outcomes.^20,21^ We have seen a marked uptake in the use of 5-ALA and intra-operative neuro-monitoring since August 2018. This would have been more difficult to achieve through an emergency-based admission pathway and has the potential to improve the extent of resection,^22,23^ minimize post-operative complications,^24^ as well as improve overall survival.^23,24^ During our study period, no significant routine changes to post-operative care were made. Routine post-operative MRI within 72 h was introduced in our hospital in 2010, as per NICE recommendations,^17^ standard deep vein thrombosis prophylaxis is used as per national guidance,^25^ and early post-operative physiotherapy involvement facilitates early safe discharges. However, currently our anesthetic teams are introducing enhanced recovery techniques to their practice, and we are introducing a physiotherapy assessment as part of the pre-operative clinical review to ensure any extra support required, is in place pre-operatively.

Our results suggest that across six years, there is an overall difference in survival between elective and emergency admissions (*χ*^2^ = 4.977, *P* = 0.032). However, this significance was not replicated in the results when patients were separated by craniotomies and biopsies. Undoubtedly, many elements contribute to this, including an increased proportion of patients undergoing craniotomies and maximal safe resections through an elective pathway; improvement in outpatient access to neuro-oncology services as a result of service reconfiguration; better understanding of molecular prognostication and patient selection, to name but a few. The degree to which these factors individually have contributed towards survival is difficult to characterize, in our study we are limited by retrospective data collection and a lack of complete data including molecular subtypes. The UK strategic vision for the NHS long team plan, including cancer services, is aimed at developing a national strategy to integrate clinical and biological data prospectively.^26^ The Tessa Jowell BRAIN MATRIX clinical trial platform^27^ will enable researchers to collect a rich genomic, pathological, and imaging dataset to provide patients with glioma and their clinicians with a fully integrated diagnosis of their disease. The end goal of this platform is to accelerate the development and delivery of brain tumour clinical trials and provide greater access to novel targeted treatments and improved outcomes for patients, both in terms of survival and quality of life.

We report an improved EOR which is associated with the use of 5-ALA. This observation is limited by selection bias, as cases deemed suitable for 5-ALA use will also be those that are deemed suitable for maximal safe resection. However, though not assessable directly, the use of 5-ALA has positive implications for survival through improved EOR.^11,28^ We did not observe a significant effect of intra-operative neuro-monitoring on post-operative neurological complications. However, the number of cases captured in this cohort who underwent neuro-monitoring was limited by size (*n* = 31). We demonstrate that uptake of routine use of adjuncts in 2018 in our trust has correlated and most likely contributed to improved EOR. The ethos and intent for maximal safe resection have also improved EOR, as we also see EOR improve across years of treatment. Due to low numbers in previous years of patients undergoing adjunct-guided resection, it is not possible to discern the relative contributions of adjunct use against improvements otherwise expected from practice change. The significance of adjunct use of improved EOR is thus likely to be over-estimated.

The median LOS for elective procedures is significantly less than the median LOS for emergency procedures, for both craniotomies and biopsies. The marked increase in the proportion of patients admitted through an elective pathway has contributed to a reduction in median LOS across the last 6 years. We show that median LOS across all procedures combined has reduced significantly when comparing between 2014 and 2019; this is especially demonstrated as a significant reduction in median LOS for elective biopsies and elective craniotomies when comparing 2014 to 2019. All patients who undergo craniotomies should be followed up within 72 h with a post-operative MRI scan to assess for adequate resection and to rule out any operative complications, as per NICE guidelines.^14^ Planned elective theatre has allowed for early scheduling of MRIs on day one of the post-operative period and the allied health therapists (physiotherapy, occupational therapy, speech, and language therapy) can be contacted in advance to facilitate safe and efficient discharge.

Complication rates did not change significantly between patients who were admitted through elective and emergency pathways. Through 2014 to 2019, our complication rate did not show consistent improvement or deterioration (range 4.8–16.7%). Incomplete documentation from earlier years may contribute to an underestimate of complications.

The cost of a neurosurgical bed is approximately £400 per night in our trust, a conservative figure that does not take into account additional costs associated with caring for patients’ individual comorbidities. The development of clinic-based services allows patients to be discharged home from district hospitals whilst awaiting specialist review, as opposed to waiting for bed space in our unit for a direct transfer. A specified operation date allows for a pre-determined admission date. This has been effective at reducing hospital LOS for patients, thereby improving patients’ experiences through their journey of treatment, as well as reducing hospital costs and improving hospital capacity. Through our experience, we show that changing from an emergency-based service to a planned, urgent elective neuro-oncology service has led to improved access to adjunct use, resective surgery, improved surgical outcomes, and reduced hospital burden. These changes will be instrumental in the improvement of survival outcomes and disease-free progression for patients with GB. We anticipate a positive end-result with further follow-up.

### Limitations

This study was limited by a retrospective data set. The large geographical area in which oncology treatment is provided further limited our ability to collect and analyze data on patients'. As of the last date of data collection (February 2021), survival data is incomplete as a significant proportion of patients undergoing primary diagnostic procedures in 2018 and 2019 are still alive. At the point of writing, results were inherently biased against patients treated in recent years, where we see the frail first to pass away. It was felt that the evaluation of survival would, at this point, be biased. With further follow-up, we expect to see improved survival outcomes and disease-free progression for patients undergoing treatment in 2018 and 2019, based on an increased proportion of craniotomies and higher extents of resections.

Due to missing molecular information from retrospective data collection, we did not feel that we had sufficient data to combine into a multifactorial analysis for survival. We acknowledge the importance that molecular subtyping plays in today’s treatment as well as a prognostication of GBs, and felt that a comprehensive survival analysis without this information would be misleading.

We acknowledge that the introduction of surgical adjuncts, including 5-ALA and neurophysiological monitoring, are associated with a learning curve. The change in service configuration and the benefits we describe here represent a period of gradual evolution, limiting direct comparison.

## Supplementary Material

npac034_suppl_Supplementary_Table_1

npac034_suppl_Supplementary_Table_2

npac034_suppl_Supplementary_Table_Legends

## Notes

**Authorship statement.** RS, VW, and CW contributed to the conception of the study, study design, analysis, and interpretation of the results. RS, SS, and VW contributed to the data collection. All authors contributed to the manuscript preparation and have approved the final version.

## Notes

**Consent for publication.** All study data were anonymized and no consent was required for this manuscript.
