# Supplementary material for: Reconfiguration from emergency to urgent elective neurosurgery for glioblastoma patients improves length of stay, surgical adjunct use, and extent of resective surgery
Source: Neurooncol Pract. 2022 May 2;9(5):420–8. doi: 10.1093/nop/npac034 (PMC9476969; doi:10.1093/nop/npac034)
Supplement: npac034_suppl_Supplementary_Table_1 [file npac034_suppl_supplementary_table_1.docx]

**Supplementary Table 1. Thirty day readmissions, complications and further operative intervention for complication, by elective or emergency admission status. *Total count 37, total patients with complications 35. One patient had infection and post-operative bleed; one patient had post-operative bleed and re-operation.**

**** Two re-operations: one due to significant post-op residual and one had repeat biopsy due to initial inconclusive biopsy.**

|  |  | **Elective** | **Emergency** |
| --- | --- | --- | --- |
| **Total** |  | 406 | 202 |
| **30 day readmission** |  |  |  |
|  | Biopsy | 1/85 (1.2%) | 4/59 (6.8%) |
|  | Craniotomy | 18/321 (5.6%) | 12/143 (8.4%) |
| **Complications** |  |  |  |
|  | Complications | 35 (8.6%) | 18 (8.9%) |
|  | No complications | 366 (90.1%) | 182 (90.1%) |
|  | Unknown | 1(0.2%) | 2 (1.0%) |
| **Complications by count*** |  |  |  |
|  | Infection | 14 | 4 |
|  | Stroke | 11 | 6 |
|  | Post-operative haematoma | 7 | 4 |
|  | Death within 30 days | 3 | 2 |
|  | Thromboembolism | 0 | 1 |
|  | Disease progression and death during admission | 1 | 0 |
|  | Re-operation | 2** | 1 |
| **Further operation due to complications** |  |  |  |
|  | Wash-out +/- bone flap removal | 8 | 5 |
|  | Evacuation of post operative haematoma | 5 | 2 |
