# Supplementary material for: Reconfiguration from emergency to urgent elective neurosurgery for glioblastoma patients improves length of stay, surgical adjunct use, and extent of resective surgery
Source: Neurooncol Pract. 2022 May 2;9(5):420–8. doi: 10.1093/nop/npac034 (PMC9476969; doi:10.1093/nop/npac034)
Supplement: npac034_suppl_Supplementary_Table_2 [file npac034_suppl_supplementary_table_2.docx]

**Supplementary Table 2. Use of 5-ALA and intra-operative neuro-monitoring adjuncts by year, and numbers of patients undergoing elective craniotomy for resection of glioblastoma.**

|  | **2014** | **2015** | **2016** | **2017** | **2018** | **2019** |
| --- | --- | --- | --- | --- | --- | --- |
| **Total elective craniotomies** | 22 | 48 | 58 | 75 | 59 | 59 |
| 5ALA | 0 | 0 | 0 | 3 | 11 | 42 |
| Awake, speech + motor monitoring | 0 | 0 | 0 | 1 | 0 | 3 |
| Awake, speech monitoring | 0 | 0 | 0 | 0 | 2 | 1 |
| Awake, motor monitoring | 3 | 0 | 2 | 1 | 3 | 0 |
| Asleep, motor monitoring | 0 | 1 | 0 | 0 | 1 | 13 |
