# Supplementary material for: Reconfiguration from emergency to urgent elective neurosurgery for glioblastoma patients improves length of stay, surgical adjunct use, and extent of resective surgery
Source: Neurooncol Pract. 2022 May 2;9(5):420–8. doi: 10.1093/nop/npac034 (PMC9476969; doi:10.1093/nop/npac034)
Supplement: npac034_suppl_Supplementary_Table_Legends [file npac034_suppl_supplementary_table_legends.docx]

**Supplementary Table legends**

Supplementary Table 1. Thirty day readmissions, complications and further operative intervention for complication, by elective or emergency admission status. *Total count 37, total patients with complications 35. One patient had infection and post-operative bleed; one patient had post-operative bleed and re-operation.

** Two re-operations: one due to significant post-op residual and one had repeat biopsy due to initial inconclusive biopsy.

Supplementary Table 2. Use of 5-ALA and intra-operative neuro-monitoring adjuncts by year, and numbers of patients undergoing elective craniotomy for resection of glioblastoma.
